# A new species and new records species of *Pluteus* from Xinjiang Uygur Autonomous Region, China

**DOI:** 10.7717/peerj.14298

**Published:** 2022-11-21

**Authors:** Zheng-xiang Qi, Ke-qing Qian, Jia-jun Hu, Yang Wang, Dong-mei Wu, Neng Gao, Pei-Song Jia, Zhen-hao Zhang, Bo Zhang, Yu Li

**Affiliations:** 1 Engineering Research Center of Edible and Medicinal Fungi, Ministry of Education, Jilin Agricultural University, Changchun, Jilin, China; 2 Joint Laboratory of International Cooperation in Modern Agricultural Technology, Ministry of Education, Jilin Agricultural University, Changchun, Jilin, China; 3 College of Biology and Food Engineering, Huanghuai University, Zhumadian, Henan, China; 4 Biotechnology Research Institute, Xinjiang Academy of Agricultural and Reclamation Sciences, Shihezi, China; 5 Institute of Plant Protection, Xinjiang Academy of Agricultural Sciences, Urumqi, China

**Keywords:** Distribution, New species, A new record, Phylogenetic analysis, Taxonomy

## Abstract

Xinjiang Uyghur Autonomous Region in China embraces a unique geographical and ecological environment, and the macrofungi represent a rich resource. However, few studies on the genus *Pluteus* have been reported from Xinjiang. In 2021, the macrofungal resources in Xinjiang were surveyed, and 10 specimens belonging to the genus *Pluteus* were collected. Based on the morphological study and molecular analysis, three species were recognized, *P. aletaiensis*, *P. brunneidiscus*, and *P. hongoi*. *Pluteus aletaiensis* is proposed as a new species. It is characterized by its bright yellow lamellae and stipe, brittle texture, subfusiform to vesicular pleurocystidia, with short pedicels to broadly lageniform to obtuse at apices, a hymeniderm pileipellis, containing dark brown intracellular pigment, and it grows on the ground. *Pluteus brunneidiscus*, a new record to China, is characterized by uneven, smooth, grayish brown to brown pileus, with an entire margin, and pointed or flatter apices intermediate cystidia, without apical hooks. *Pluteus hongoi*, a new record to Xinjiang Uyghur Autonomous Region, China, is characterized by the apical hook’s structure (commonly bifid) of pleurocystidia. The nuclear internal transcribed spacer (nrITS) and translation elongation factor 1-alpha (TEF1-a) region were used for the molecular analysis. Phylogenetic trees were constructed using both the maximum likelihood analysis (ML) and Bayesian inference (BI). Detailed descriptions of the three species are presented herein. Finally, a key to the list of eight species of the genus *Pluteus* knew from Xinjiang is provided.

## Introduction

Genus *Pluteus* (Fr.) Quél. was established by Fries in 1836 and belongs to Basidiomycota, Hymenomycetes, Agaricales, Pluteaceae. The genus is distinguished from other agarics by the main features of the free and white, pink or pinkish brown lamellae, a pink spores print, mostly grow on rotten wood; smooth, non-dextrinoid, inamyloid, and pale pink basidiospores, divergent lamellar trama, thick- or thin-walled pleurocystidia (Vellinga & Schreurs, 1985; Singer, 1986; Justo et al., 2011a, 2011b).

Early studies (Lange, 1917; Imai, 1938; Singer, 1956), based on features such as pleurocystidia and pileipellis, divided the genus *Pluteus* into three sections: (1) section *Pluteus* Fr was characterized by pileipellis a cutis and thick-walled pleurocystidia, (2) section *Hispidoderma* Fayod was characterized by pileipellis a trichoderm composed of elongated cells and thin-walled pleurocystidia, and (3) section *Celluloderma* Fayod was characterized by pileipellis a hymeniderm or hymeniderm with cystidioid elements that consists of clavate to spheropedunculate cells and thin-walled pleurocystidia. However, other taxonomists held different perspectives. Kühner (1926, 1980) divided the genus *Pluteus* into two sections, one section with pileipellis a cutis and thick-walled pleurocystidia, and the other section with thin-walled pleurocystidia; Vellinga & Schreurs (1985), based on the characteristics of the pileipellis and pleurocystidia, divided the genus *Pluteus* into three sections: section *Pluteus*, section *Villosi* Vellinga & Schreurs, and section *Celluloderma*. Section *Pluteus* with pileipellis as a cutis and thick-walled pleurocystidia; section *Villosi* with pileipellis as a trichoderm and thin-walled pleurocystidia; section *Celluloderma* with a hymeniderm pileipellis and thin-walled pleurocystidia. In later studies, Singer (1958, 1986) divided section *Celluloderma* into two subsections based on the shape of pileipellis. (1) Subsection *Mixtini* Singer characterized by pileipellis as a hymeniderm with cystidioid elements that consists of two types of cells (ellipsoidal, balloon-shaped to inverted pyriform cells as well as elongated cells); (2) subsection *Eucellulodermini* Singer characterized by pileipellis as a hymeniderm that consists of cystidia-like cells. In addition, later Kobayashi (2002) established a new section-section *Horridus* S. Ito & S. Imai, including one species, *P. horridilamellus* S. Ito & S. Imai. Recently, based on the analyses of combined nSSU, ITS, and nLSU datasets, the genus *Pluteus* has been subdivided into three major lineages: (1) section *Pluteus* contains taxa with metuloid pleurocystidia and a pileipellis as a cutis, (2) section *Hispidoderma* are characterized by a pileipellis composed of elongated elements, very variable in shape and size, organized as a hymeniderm or trichoderm. (3) section *Celluloderma* includes species with non-metuloid pleurocystidia and a pileipellis as an euhymeniderm or an epithelioid hymeniderm composed of short elements, intermixed or not with elongate cystidioid elements and species with a cutis-like pileipellis and non-metuloid cystidia. The species with cutis-like pileipellis and possessing a partial veil, formerly placed in the genus *Chamaeota* (W.G. Sm.) Earle, are also included in *Pluteus* section *Celluloder*ma (Justo et al., 2011b).

According to reports, about 300 species of the genus *Pluteus* were reported worldwide (Kirk et al., 2008), but only about 50 species were reported from China, distributed in 25 provinces or autonomous regions (Xu, 2016; Yang & Bau, 2010). In China, Patouillard (1893) was the first to report *Pluteus* species, then Chow (1935) was the first Chinese to studies on the genus *Pluteus* in China. Bi, Zheng & Li (1993) recorded 10 species of the genus in *The macrofungus flora of China’s Guangdong Province*; Redhead & Liu (1982) wrote a new species of *p. luteus* (Redhead & Liu, 1982) Redhead from China, now a synonym of *p. variabilicolor* Babos (Ševčíková & Dima, 2021). Xie, Wang & Wang (1986) recorded seven species of the genus *Pluteus* in the Changbai Mountain area in *Illustrations of Agarics from Changbai Mountains*, Mao (2000) recognized 12 species of genus *Pluteus* in China in the *Macrofungi in China*, Li & Bau (2003) reported five species of genus *Pluteus* in Changbai Mountain area in *Mushrooms of Changbai mountain in China*; Zhuang et al. (2005) included 15 species of genus *Pluteus* in *Fungi of Northwestern China*. In recent years, many new and newly recorded species in China have also been published (Xi et al., 2011; Hosen et al., 2018, 2019, 2021).

Although the Xinjiang Uyghur Autonomous Region has a unique geographical and ecological environment, only five *Pluteus* species have been reported from Xinjiang, and many resources need to be clarified. *P. thomsonii* (Berk. & Broome) Dennis. was reported from the Jengish Chokusu (Mao, Wen & Sun, 1978). Wang & Zhao (1997) reported *P. cervinus* (Schaeff.) P. Kumm. from the Central Tianshan Forest Region. Mao (2000) recorded *P. umbrosus* (Pers.) P. Kumm. from Xinjiang. Zhao (2001) recognized *P. cervinus*, *P. leoninus* (Schaeff.) P. Kumm., and *P. pellitus* (Pers.) P. Kumm. from Xinjiang.

We conducted a preliminary investigation of the taxonomy of *Pluteus* species in Xinjiang. The goal of the present study was to provide an annotated list of all species recorded, describe one new species, list newly recorded species from China and newly recorded species from Xinjiang, give detailed descriptions and illustrations of three species, and clarify the phylogenetic relationships of the revealed species and related taxa from the genus *Pluteus* based on morphological and molecular studies.

## Materials and Methods

### Specimens and morphological description

#### Site description

Xinjiang Uygur Autonomous Region is located in the hinterland of the Eurasian continent of northwestern China. For example, there are distinctive landforms, including the world’s second-highest peak. It is surrounded by high mountains, resulting in early dryness and precipitation varies significantly among regions. There are continental cold temperate zone cold climate, temperate zone continental monsoon climate, temperate zone continental arid climate, and alpine climates from north to south (Yao et al., 2022).

**Figure 1:**
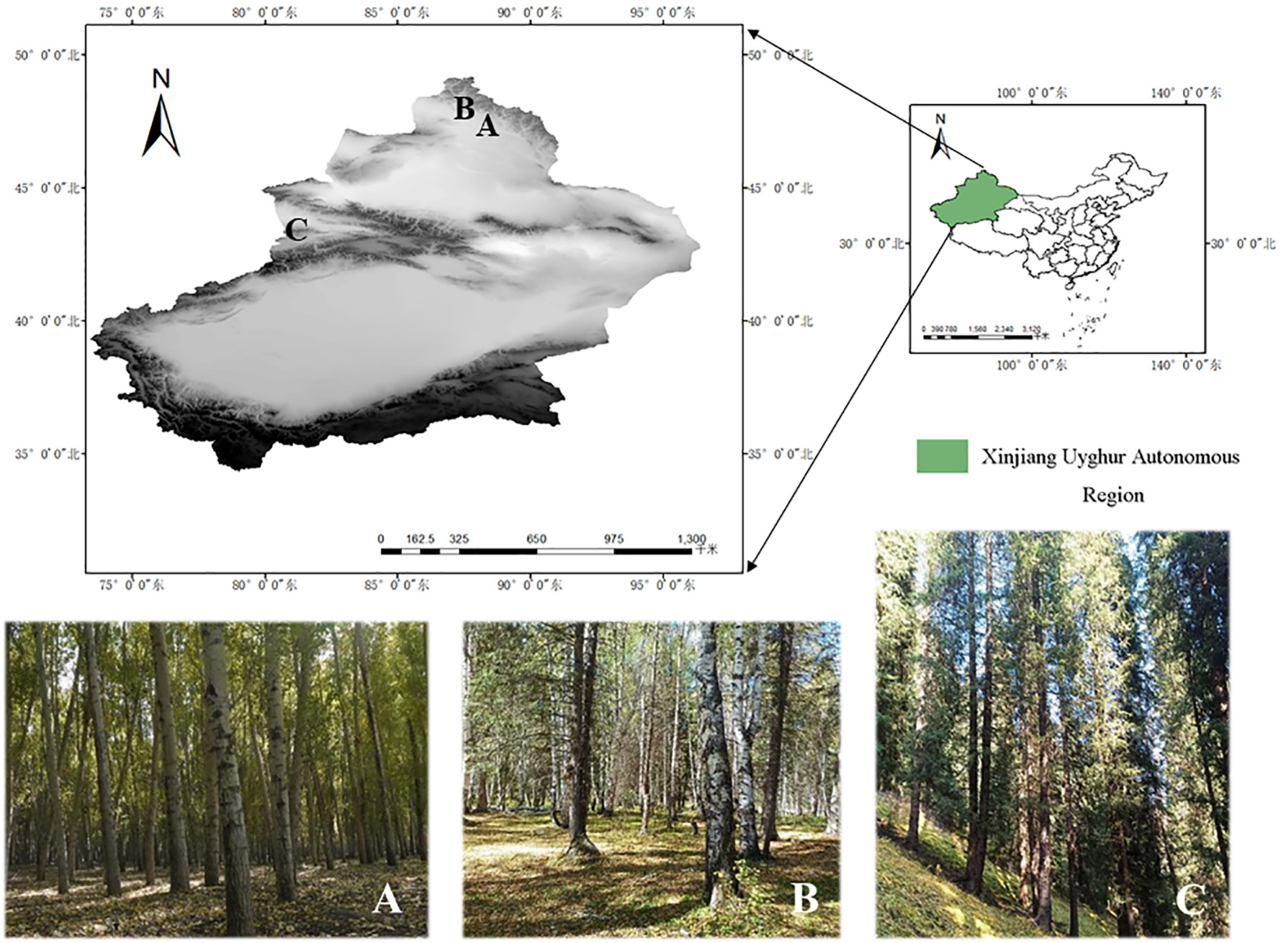
Specific collection sites for three species. (A) Habitat/landscape for *Pluteus aletaiensis*. (B) Habitat/landscape for *P. hongoi*. (C) Habitat/landscape for *P. brunneidiscus*.

In this study, the specimens of the genus *Pluteus* were collected from Kelan River Valley in Altay City (47°50′14.66″N, 88°13′23.41″E). Altay Region with an altitude of about 577 m, Wolong Bay in Kanas (48°65′77.01″N, 87°03′82.11"E). Altay Region with an altitude of 1,333 m, and Shuimogou in Regiment 74 (43°12′50.69″N, 80°13′18.44″E). Zhaosu County, Ili Kazakh Autonomous Prefecture with an altitude of 1,764 m. See Fig. 1 for details.

### Collection and morphological studies

Photos of fresh basidiomata were taken in the field, scientifically fully reflecting the growth environment and the characters, including the shape of the pileus, the color of lamellae, and color codes followed by Munsell Soil Color Charts (Munsell, 2009). The size of basidiomata was measured when fresh, and detailedly recorded the macroscopic characteristics, such as shape, size, and color of the pileus, odor, color, and density of lamellae. A small portion of fresh context and lamellae was dried on silica gel and used for DNA extraction. Fresh basidiomata were dried at 40–50 °C using an electric drier and preserved at the Herbarium of Mycology of Jilin Agricultural University (HMJAU). The observation of microstructure characteristics were based on dry specimens. The dry specimens were rehydrated in 94% ethanol for microscopic examination and then mounted in 3% potassium hydroxide (KOH), 1% Congo Red, and Melzer’s Reagent, using a light microscope (ZEISS, DM1000, Oberkochen, Germany). The morphological descriptions follow Largent, Johnson & Watling (1977). As previously described in Rao et al. (2021b), data were collected. Specifically, the following symbols were used in the description: [n/m/p] indicates that ‘n’ randomly selected basidiospores from ‘m’ basidiomata of ‘p’ collections were measured, ‘avl’ means the average length of basidiospores, except for the extreme values, ‘avw’ refers to the average width of the basidiospores, except the extreme values, ‘Q’ represents the quotient of the length and width of a single basidiospore inside view, ‘Qm’ refers to the average Q value of all basidiospores ± standard deviation. Dimensions for basidiospores are given as (a)b–c(d). The range of b–c contains a minimum of 90% of the measured values. Extreme values (*i.e.*, a and b) are given in parentheses.

### Research methods of molecular systematics

#### DNA extraction, PCR amplification, and sequencing

The total DNA of the specimens was extracted by the new plant genomic DNA extraction kit from Jiangsu Kangwei Century Biotechnology Limited Company. The amplification primers of the ITS nrDNA (ITS) regions were ITS1 and ITS4 (White et al., 1990), and TEF1-α regions were EF1- 983F and EF1-1567R (Rehner & Buckley, 2005). The amplification reactions were carried out in a 25 μl system. The total amount of PCR mixed was as follows: dd H_2_O 13.5 μl, 10 × Taq Buffer 5 μl, 10 mM dNTPs 1 μl, 10 mM upstream primer 1 μl, 10 mM downstream primer 1 μl, DNA sample 2 μl, 2 U/μm Taq Polymerase 1.5 μl. The cycle parameters were as follows: 4 min at 94 °C; 30 s at 94 °C, 40 s at 53 °C, 1 min at 72 °C for 36 cycles; 10 min at 72 °C; storage at 4 °C (Xu, 2016). The PCR product was subjected to 1% agarose gel electrophoresis. The purified PCR products were sent to Sangon Biotech Limited Company (Shanghai, China) for sequencing using the Sanger method. The sequencing results were clipped with Seqman 7.1.0 (Swindell & Plasterer, 1997) and then submitted to GenBank (https://www.ncbi.nlm.nih.gov/genbank/).

#### Data analysis

Based on BLAST results, morphological similarities, and the related articles (Menolli, Asai & Capelari, 2010; Justo et al., 2014; Menolli, Justo & Capelari, 2015; *Rao et al., 2021a;* Ševčíková et al., 2022), ITS and TEF1-α sequences obtained and related to these samples are listed in Table 1. The ITS and TEF1-α dataset comprised 106 representative sequences showing the highest similarity to *Pluteus* spp., and two sequences of *Volvopluteus michiganensis* (A.H. Sm.) Justo & Minnis. as an outgroup.

**Table 1:** ITS + TEF1-α sequence information for phylogenetic analysis.

Taxon	Collection	Country	nrITS	TEF1α
*pluteus alniphilus*	–	France	KJ009677	–
*p. alniphilus*	–	Russia	KJ009676	–
*P*. aff. *romellii*	AJ 215	Spain	HM562054	ON813269
*P*. aff. *romellii*	BRNM 792987	Czech Republic	ON864083	ON813272
*P*. aff. *romellii*	LB 15121104	Spain	ON864082	ON813271
*P*. aff. *romellii*	LE 312975	Russia	ON864081	ON813270
*P*. aff. *romellii*	LE 313340	Russia	ON864084	ON813273
*P. aurantiorugosus*	GDGM41547	China	MK791275	–
*P. aurantiorugosus*	GM 2580	Spain	ON864101	–
*P. aurantiorugosus*	LE 312803	Russia	ON864105	–
*P. aurantiorugosus*	LE 312815	Russia	ON864103	ON813296
*P. aurantiorugosus*	LE 313555	Russia	ON864104	–
*P. aurantiorugosus*	Voucher 880	Italy	JF908608	–
*P. aurantiorugosus*	Voucher 2847	Italy	JF908613	–
*P. aureovenatus*	SP 393697	Brazil	FJ816663	KJ010056
*P. aureovenatus*	SP 394388	Brazil	HM562160	–
*P. aureovenatus*	SP 416735	Brazil	KM983702	–
*P. austrofulvus*	AJ 857	USA	KM983701	ON813290
*P. austrofulvus*	AJ 860	USA	KM983699	ON813288
*P. austrofulvus*	iNaturalist 112016967	USA	ON864095	ON813291
*P. austrofulvus*	iNaturalist 112219822	USA	ON864096	ON813292
*P. austrofulvus*	iNaturalist 112280046	USA	ON864097	ON813293
***p. aletaiensis***	**HMJAU 60207**	**China**	**OM991943**	OP573273
***p. aletaiensis***	**HMJAU 60208**	**China**	**OM992247**	OP573274
***p. aletaiensis***	**HMJAU 60209**	**China**	**OM992249**	OP573275
*p. brunneidiscus*	–	Canada	KJ009691	–
*p. brunneidiscus*	–	Canada	KJ009692	–
***p. brunneidiscus***	**HMJAU 60206**	**China**	**OM991893**	–
***p. brunneidiscus***	**HMJAU 60210**	**China**	**OM943513**	–
*P. castaneorugosus*	LE 313071	Vietnam	MT611237	–
*p. cervinus*	–	Russia	KJ009629	–
*p. cervinus*	–	Russia	KJ009632	–
*P. exilis*	–	USA	KJ009659	–
*P. fulvibadius*	AJ 815	USA	KM983698	ON813285
*P. fulvibadius*	HRL3391	Canada	ON864094	ON813287
*P. fulvibadius*	MO 270623	USA	ON864093	ON813286
*P. globiger*	ICN139025	Brazil	JQ065030	–
*p. hongoi*	–	Japan	HM562100	–
*p. hongoi*	–	Japan	HM562101	–
***p. hongoi***	**HMJAU 60205**	**China**	**OM302007**	–
*P. iguazuensis*	NK I10	Brazil	KM983704	–
*p. kovalenkoi*	–	Russia	KJ009697	–
*P. pallescens*	K (M) 93678	UK	ON864073	–
*P. parvicarpus*	LE 313357	Russia	ON864114	ON813302
*P. parvicarpus*	LE 313631	Russia	ON864115	ON813303
*P. parvisporus*	AJ 855	USA	ON864099	ON813295
*P. parvisporus*	iNaturalist 112236342	USA	ON864098	ON813294
*P. paucicystidiatus*	SP 394383	Brazil	HM562173	–
*P. pauperculus*	JAC11068	New Zealand	MN738636	–
*P. pauperculus*	JAC9790	New Zealand	MN738621	–
*P. phlebophorus*	AJ81	Spain	HM562039	ON133554
*P. romellii*	AJ 232	Spain	HM562062	ON813280
*P. romellii*	BRNM 761731	Czech Republic	ON864065	ON813278
*P. romellii*	BRNM 816205	Czech Republic	ON864063	ON813276
*P. romellii*	BRNM 817530	Slovakia	ON864072	ON813282
*P. romellii* f. *albidus*	MCVE 28336	Italy	KM035790	–
*P. romellii* var. *luteoalbus*	BRNM 788199	Czech Republic	LT838190	–
*P. rugosidiscus*	BRNM761706	Slovakia	MH010876	LT991752
*P. rangifer*	–	Russia	KJ009651	–
*P. rangifer*	–	Russia	KJ009654	–
*p. salicinus*	–	Spain	HM562051	–
*p. salicinus*	–	Spain	HM562174	–
*p. saupei*	–	USA	HM562113	–
*p. shikae*	–	Japan	HM562095	–
*p. shikae*	–	Russia	KJ009696	–
*P. siccus*	LE 313356	Russia	ON864113	ON813301
*P. stenotrichus*	AJ 352	Dominican Republic	JN603201	–
*P. sternbergii*	PRM 154258	Czech Republic	ON864116	–
*P. sublaevigatus*	SP 393694	Brazil	FJ816667	–
*Pluteus* sp.	AJ 842	Dominican Republic	KM983705	–
*Pluteus* sp.	SP 416739	Brazil	KM983703	–
*P. vellingae*	BRNM 817769	Czech Republic	ON864108	ON813297
*P. vellingae*	ECV 3201	USA	AY854065	AY883433
*P. vellingae*	FG02092019008	Slovenia	ON864112	ON813299
*P. vellingae*	GM 3260	Spain	ON864107	ON813298
*P. vellingae*	OKA-TR512	Turkey	–	ON813300
*Volvopluteus michiganensis*	–	China	MW242664	–
*V. michiganensis*	–	China	MW242665	–

**Note:**

Bold fonts are the sequences to be determined in this study.

For the ITS dataset, the ‘auto’ strategy and normal alignment mode of MACSE V2.03 (Ranwez et al., 2018) and MAFFT (Katoh & Standley, 2013) were used for sequence alignment, then manually adjusted in BioEdit v7.1.3 (Hall, 1999). ModelFinder (Kalyaanamoorthy et al., 2017) selected the best-fit models using the Bayesian information criterion (BIC). The maximum likelihood (ML) analyses were performed in IQTree 1.6.8 (Nguyen et al., 2015), and the Bayesian inference phylogenies were performed in MrBayes 3.2.6 (Ronquist et al., 2012) (two parallel runs, 2,000,000 generations), in which the initial 25% of sampled data were discarded as burn-in. The above software was integrated into PhyloSuite 1.2.2 (Zhang et al., 2020). The ML phylogenetic tree was evaluated using the bootstrap method with a bootstrap value of 1,000 replicates; BI determined that the analysis reached smoothness with variance <0.01 and terminated the calculation. The evolutionary tree was followed up with Figtree v1.4.

For the ITS + TEF1-α dataset, sequence alignment was performed for ITS and TEF1-α using the "automatic" strategy of MACSE V2.03 (Ranwez et al., 2018) and MAFFT (Katoh & Standley, 2013) and normal alignment mode, respectively, and then manually adjusted in BioEdit v7.1.3 (Hall, 1999). Afterward, ITS and TEF1-α sequences were combined using PhylosuitV1.2.2 (Zhang et al., 2020). ModelFinder (Kalyaanamoorthy et al., 2017) selected the best-fit models using the Bayesian information criterion (BIC). The maximum likelihood (ML) analyses were performed in IQTree 1.6.8 (Nguyen et al., 2015), and the Bayesian inference phylogenies were performed in MrBayes 3.2.6 (Ronquist et al., 2012) (two parallel runs, 2,000,000 generations), in which the initial 25% of sampled data were discarded as burn-in. The above software was integrated into PhyloSuite 1.2.2 (Zhang et al., 2020). The ML phylogenetic tree was evaluated using the bootstrap method with a bootstrap value of 1,000 replicates; BI determined that the analysis reached smoothness with variance <0.01 and terminated the calculation. The evolutionary tree was followed up with Figtree v1.4.

### Nomenclature

The electronic version in Portable Document Format (PDF) will represent a published work according to the International Code of Nomenclature for algae, fungi, and plants. Hence the new names contained in the electronic version are effectively published under that Code from the electronic edition alone. In addition, new names contained in this work have been submitted to MycoBank from where they will be made available to the Global Names Index. The unique MycoBank number can be resolved and the associated information viewed through any standard web browser by appending the MycoBank number contained in this publication to the prefix “http://www.mycobank.org/MycoTaxo.aspx?Link=T&Rec=.”. The online version of this work is archived and available from the following digital repositories: PeerJ, PubMed Central, and CLOCKSS.

## Results

### Phylogenetic analyses

**Figure 2:**
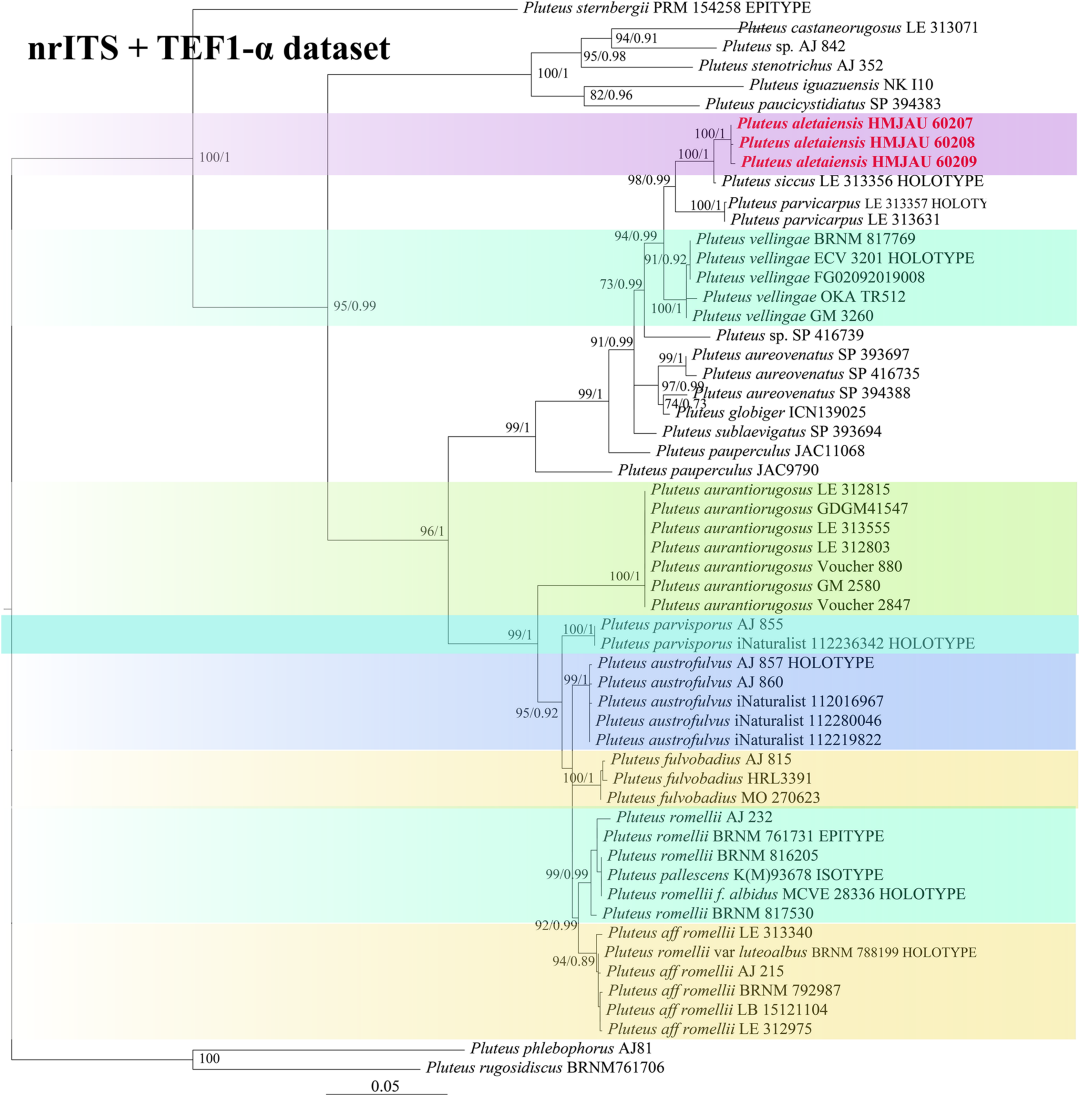
Phylogenetic tree of the section *Celluloderma* of the genus *Pluteus*. Best tree from the ML and BI analysis of the nrITS + TEF1-α dataset. The two values of internal nodes respectively represent maximum likelihood bootstrap (MLBP)/Bayesian posterior probability (BIPP). In at least two analyses, the thick node indicates the significantly-supported branch (MLBP ≥ 70, BIPP ≥ 95%). The collection number is marked after the species name. New species is indicated in bold and red font.

**Figure 3:**
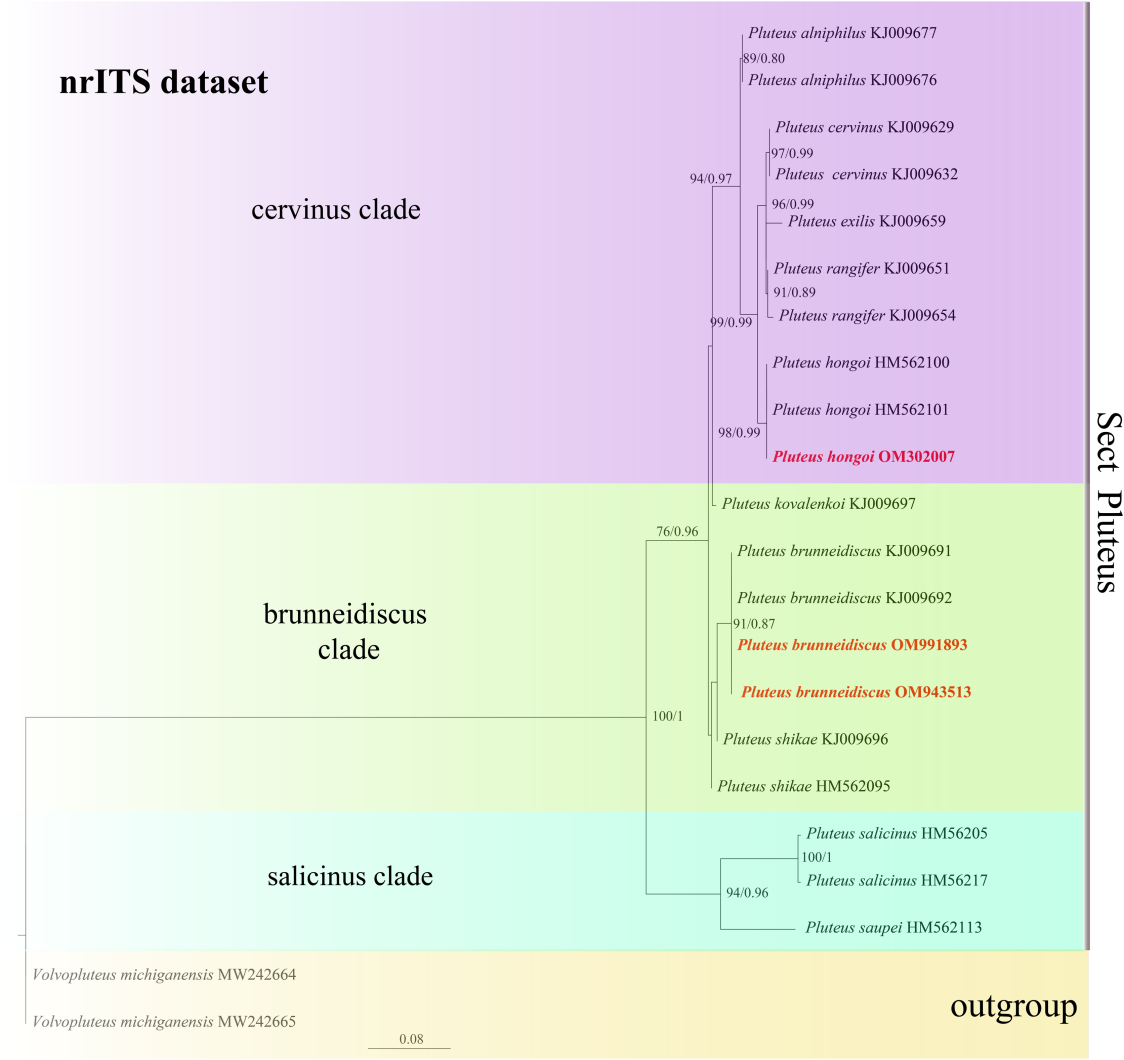
Phylogenetic tree of the section *Pluteus* of the genus *Pluteus*. Best tree from the ML and BI analysis of the nrITS dataset. The two values of internal nodes respectively represent maximum likelihood bootstrap (MLBP)/Bayesian posterior probability (BIPP). In at least two analyses, the thick node indicates the significantly-supported branch (MLBP ≥ 70, BIPP ≥ 95%). The GenBank accession number is marked after the species name. Two species from China are expressed in bold and red font, and *Volvopluteus michiganensis* is selected as the outgroup.

In the dataset, 111 sequences derived from two gene loci (ITS and TEF1-α) from 35 samples were used to build phylogenetic trees; nine of these were newly generated, with six ITS sequences and three TEF1-α sequences. The phylogenetic construction performed *via* ML and BI analysis for the two combined datasets showed a similar topology (Figs. 2 and 3).

In the present study, six sequences of *P. aletaiensis* were gathered into a single branch along with high support (100/1). *Pluteus aletaiensis* is classified into the section *Celluloderma*, and sisters to *P. siccus* E.F. Malysheva, *P. parvicarpus* E.F. Malysheva, *P. vellingae* Justo, Ferisin, Ševčíková, Kaygusuz, G. Muñoz, Lebeuf & S.D. Russell, *P. sublaevigatus* (Singer) Menolli & Capelari and *P. globiger* Singer,. with a very high support rate (Fig. 2). The sequence of *P. brunneidiscus* and *P. hongoi* from Xinjiang Uygur Autonomous Region were gathered into the sect. *Pluteus* and formed independent clades with high support (Fig. 3).

### Taxonomy

***Pluteus aletaiensis* Z.X. QI, B. Zhang & Y. Li, sp. nov.**

MycoBank No. MB 840297

(Figs. 4A–4E and 5)

**Figure 4:**
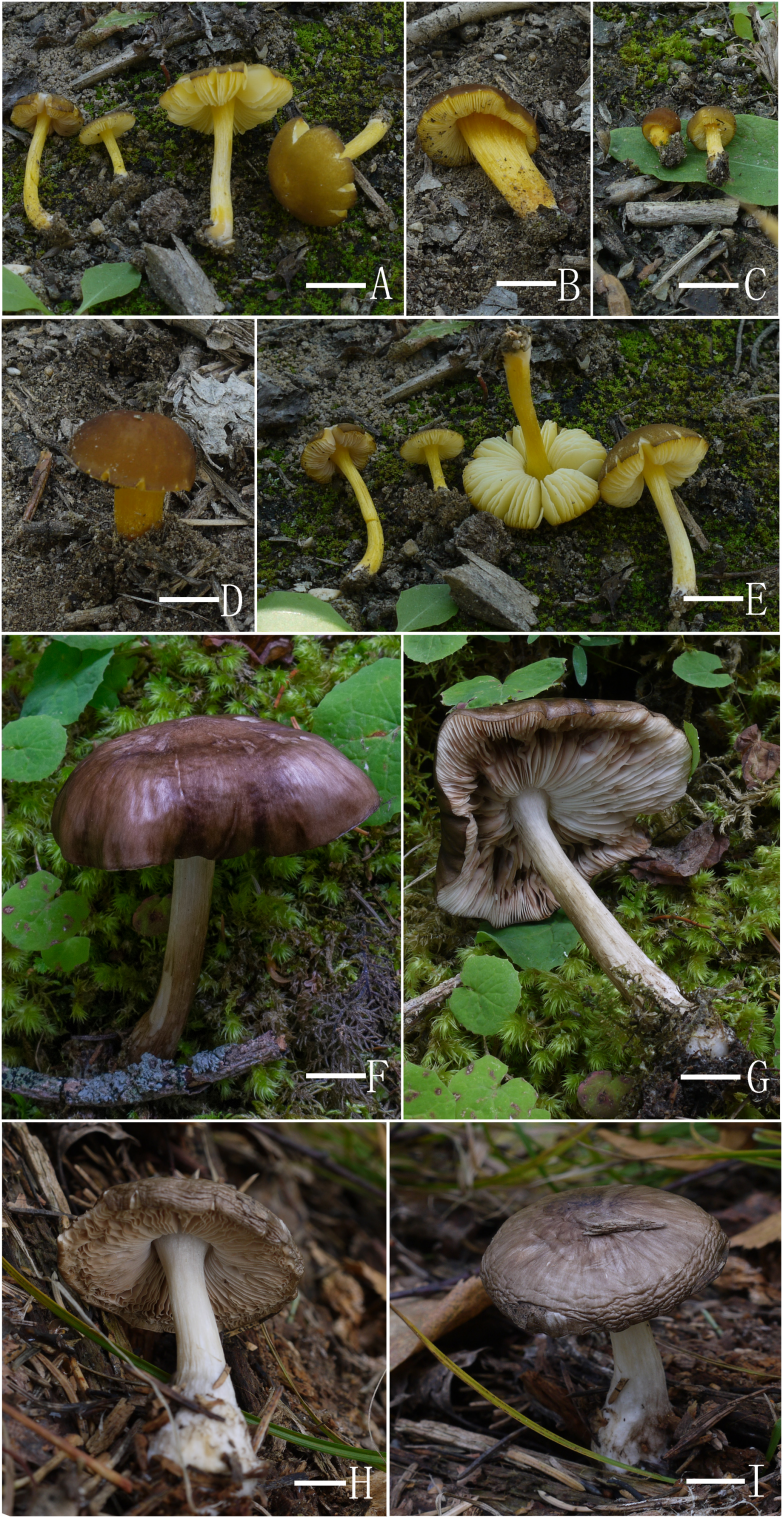
Basidiomata features. (A–E) *Pluteus aletaiensis*. (F–G) *P. brunneidiscus*. (H–I) *P. hongoi*. (A–I) Photos by Zheng-xiang Qi. Scale bars: 1 cm.

**Figure 5:**
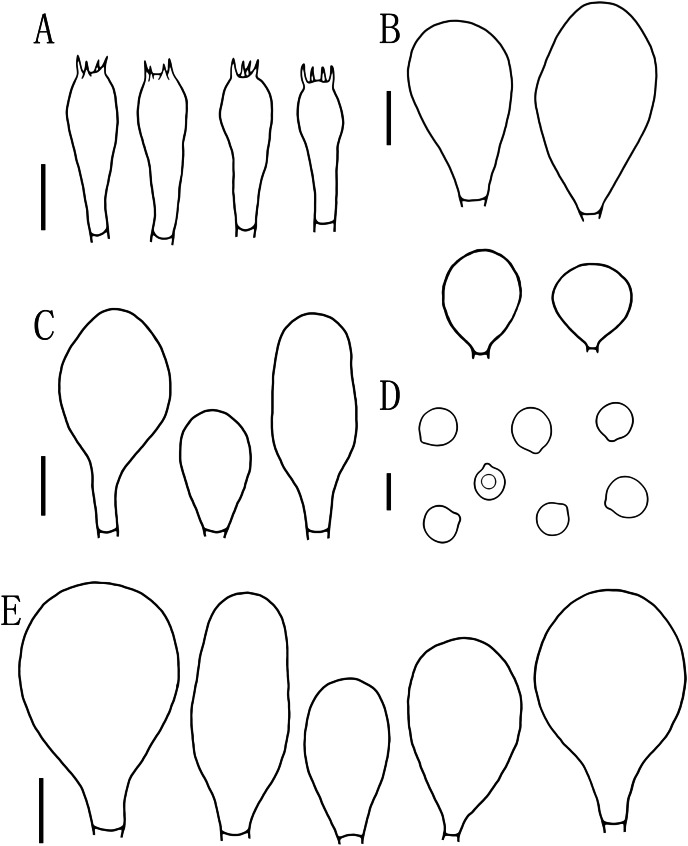
Microscopic features of *Pluteus aletaiensis*. (A) Basidia. (B) Pileipellis. (C) Cheilocystidia. (D) Basidiospores. (E) Pleurocystidia. Scale bars: (D) 5 µm; (A, B, C, E) 10 µm.

**Etymology.** The epithet “Aletaiensis” refers to the Aletai Region, the location of the holotype.

**Holotype.** CHINA. Xinjiang Uygur Autonomous Region, Aletai Region, Aletai City, Cran River Valley, 47°50′14.66″N, 88°13′23.41″E, ASL 577 m, 14 September 2021, Z.X. Qi (HMJAU 60207), GenBank: OM992247.

**Diagnosis.** *Pluteus aletaiensis* differs from *P. romellii* by its bright brownish yellow, dark brownish-yellow in the middle of pileus, surface cover with white punctuations stipe, brittle texture. Microscopically, narrower pleuro- and cheilo-cystidia, a hymeniderm pileipellis, with globose to short-clavate, containing dark brown intracellular pigment.

**Description**

Basidiomata tiny to small size. Pileus 5–22.5 mm broad, initially paraboloid to hemispherical, later oblong hemispherical with an umbo, brownish-yellow when young (2.5YR 4/10), bright brownish yellow at maturity (7.5Y 6/12-7.5Y 8/14), dark brownish-yellow in the middle (7.5Y 5/8), not striated, with varying degrees of dehiscence at the margin at maturity. Context yellow, 0.5–1 mm thick. Lamellae close, bright yellow to yellowish (2.5Y 8/6**-**2.5Y 9/8), edges white (2.5Y 9/6), free, unequal, ventricose, 2–6 mm wide, entire. Stipe 4.5–25 mm long and 1.5–3 mm wide, central, slender, clavate, slightly thick and slightly curved at base, brittle, bright yellow to yellowish (2.5Y 8/6**-**2.5Y 9/8), with longitudinal striation, white punctuations, and white pruinose mycelium at the base. Spore print unknown.

Basidiospores [100, 12, 3] (5.5)6.0–7.5(8.0) × 5.5–6.5 (7) μm, avl = 7.0 μm, avw = 6.0 μm, Q = (1.08)1.09–1.20(1.24), Qm = 1.16 ± 0.05, globose, subglobose to broadly elliptical, slightly pinkish, thick-walled, smooth, non-dextrinoid, partially containing one droplet or irregular inclusions. Basidia 27–43 × 7–12(13) μm, fusiform to clavate, usually 4-sterigmate, 2–3 sterigmate occasional, thin-walled, and hyaline in KOH. Pleurocystidia 51–75(80) × (11)13–22(23) μm, abundance, subfusiform to vesicular, with short pedicels to broadly lageniform to obtuse at apices, thin-walled, and hyaline in KOH. Cheilocystidia 25–40 × (11)12–20 μm, abundant, clustered, vesiculose or pyriform to broadly fusoid-ventricose with pedicels short necks, with obtuse at apices, thin-walled, and hyaline in KOH. Pileipellis a hymeniderm, cells 15–43 × 10–34(35) μm, composed of globose to short-clavate cells, thin-walled, and hyaline in KOH, containing dark brown intracellular pigment, cells tightly adhering one to another. Stipitipellis a cutis of cylindrical hyphae, elements 5–17 μm wide, thin-walled, hyaline in KOH, septate, without apparent contents. Clamp connections absent in all tissues.

**Ecology and distribution.** Scattered on the ground in the broad-leaved forest (*Populus alba* var. *pyramidalis* Bge). Known from Xinjiang Uygur Autonomous Region of China.

**Additional specimens examined.** China. Xinjiang Uygur Autonomous Region, Aletai Region, Aletai City, Cran River Valley, 47°50′14.56″N, 88°13′23.45″E, ASL 580 m, 15 September 2021, Z.X. Qi, D.M. Wu, N. Gao & B.K. Cui, HMJAU 60208 (OM991943); Xinjiang Uygur Autonomous Region, Altay Region, Altay City, Cran River Valley, 47°50′14.69″N, 88°13′23.47″E, ASL 576 m, 16 September 2021, Z.X. Qi, D.M. Wu, N. Gao & B.K. Cui, HMJAU 60209 (OM992249).

Note. In our phylogenetic tree (Fig. 2), *P. aletaiensis* clustered with similar species of section *Celulloderma* and together with *P. siccus P. parvicarpus, P. vellingae, P. sublaevigatus*, and *P. globiger* with high support (Fig. 2). On phylogenetic trees, *P. siccus* display a close relationship to *P. aletaiensis*; morphologically, they are distinctly different, *P. siccus* (Ševčíková et al., 2022) is distinguishable from *P. aletaiensis* due to its slightly velvety pileus with a greenish hue, and smaller basidiospores (about 4.6–6.0 × (3.5–)4.5–5.5 µm), the polymorphic cheilocystidia, and grows on decaying wood and geographically distributed in the Russian Far East. *P. parvicarpus* (Ševčíková et al., 2022) could be distinguished from *P. aletaiensis* by its sulcate-striate at the margin of pileus, smaller basidiospores (about 4.5–6.0 × 4.2–5.5 µm), solitary on fallen branches of deciduous trees, and geographical distribution in the Russian Far East. *P. aletaiensis* and *P. vellingae* (Ševčíková et al., 2022) are both with yellow-brown to brown pileus and similar basidiospores. *P. vellingae* has broadly clavate to clavate or ovoid pleurocystidia, and it grows on coniferous or deciduous wood. *P. sublaevigatus* (Menolli, Asai & Capelari, 2010) differs from *P. aletaiensis* with its slightly rugulose at the center of the pileus, and translucently striate at the margin, free to subfree lamellae with lamellulae, and gregarious on decaying wood. *P. aletaiensis* and *P. globiger* (Singer & Digilio, 1952; Dias & Cortez, 2013) are both smaller pileus (diameter not more than 23 mm), but the lamellae of *P. aletaiensis* are bright yellow to yellowish, while *P. globiger* are greyish orange; on the other hand, *P. globiger* has ventricose of the cheilocystidia.

Morphologically, we excluded some relatively names species that are highly similar to *P. aletaiensis*, such as *P. fulvibadius* Murrill, *P. romellii* (Britzelm) Lapl., *P. sternbergii* Velenovský, and *P. sulphureus* Velenovský.

*P. fulvibadius* Murrill is also similar to *P. aletaiensis. P. fulvibadius*, which is widely distributed in the United States, has been reported by many mycologists (Murrill, 1917; Minnis, 2008; Minnis & Sundberg, 2010). *P. fulvibadius* differs from *P. aletaiensis* by its bigger pileus, which reaches 15–50 mm broad, slight to strongly rugose around the center, and translucently striate at the margin. In microfeatures, the pleurocystidia of *P. fulvibadius* are cylindrical-clavate; the pleurocystidia and the cheilocystidia are covered with an apical cap of amorphous mucilaginous material (Minnis, 2008; Minnis & Sundberg, 2010; Ševčíková et al., 2022).

*P. romellii* is easily confused with *P. altaiensis* due to its highly similar morphology, and a synonym of *P. romellii* is *P. lutescens* (Singer, 1956; Vellinga & Schreurs, 1985; Orton, 1986; Minnis, 2008). However, the pileus of *P. aletaiensis* is oblong hemispherical with an umbo, bright brownish yellow, dark brownish-yellow in the middle, while the color of *P.romellii* variable (date-brown, umber, or snuff-brown), glabrous, venation-like protrusions, and with translucent-striate at the margin; on the other hand, pleuro- and cheilo-cystidia of *P. romellii* are broader (52–62 × 14–30 μm and 23–61 × 12–36 μm respectively) than *P. altaiensis* (51–75(–80) × (11–)13–22(–23) μm and 25–40 × (11–)12–20 μm respectively) (Singer, 1956; Orton, 1986; Vellinga, 1990; Minnis, 2008). In the phylogenetic tree, *P. romellii* and *P. altaiensis* are not clustered in the same branch.

*Ševčíková et al*. obtained the molecular sequence of *P. sternbergii* (ON864116) and verified the position of *P. sternbergii* in the phylogenetic tree, not in the romellii clade, but in the cinereofuscus clade, therefore, selected collection PRM 154258 as the epitype of *P. sternbergii*. (Ševčíková et al., 2022). Morphologically, the basidiospores, basidia, pleurocystidia, and stipitipellis of *P. aletaiensis* and *P. sternbergii* are similar, while *P. sternbergii* grows in the stump of Populus.

For *P. sulphureus*, Ševčíková considers the specimens preserved in herbarium PRC (Velenovský no. 95!) as not belonging to the genus *Pluteus* (Ševčíková et al., 2022).

*Pluteus brunneidiscus* Murrill, N. Amer. Fl. (New York) 10(2): 131 (1917).

Syn.: *Pluteus washingtonensis* Murrill, N. Amer. Fl. (New York) 10(2): 135 (1917).

(Figs. 4F–4G and 6)

**Figure 6:**
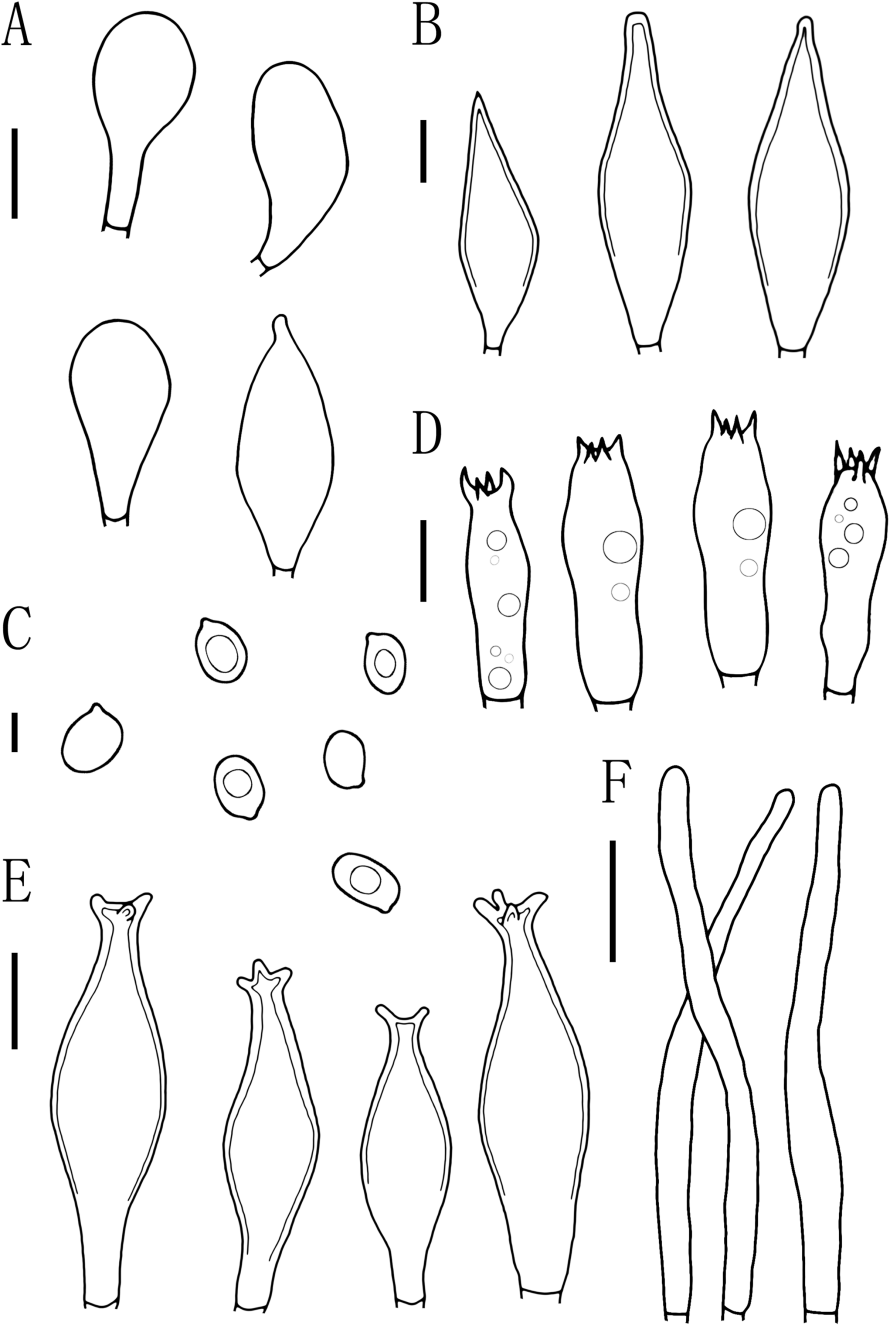
Microscopic features of *Pluteus brunneidiscus*. (A) Cheilocystidia. (B) Intermediate cystidia. (C) Basidiospores. (D) Basidia. (E) Pleurocystidia. (F) Pileipellis. Scale bars: (C) 5 µm; (B, D) 10 µm; (A, E) 20 µm; (F) 40 µm.

**Description**

Basidiomata large-sized. Pileus 68 mm broad, surface shiny and smooth, grayish brown to brownish brown (10.0YR 4/10**-**10.0YR 3/6), margin spreading to partially collapsed, uneven, entire, partly collapsed. Lamellae close, dirty white (10.0YR 9/2), turning dark brown at maturity (5.0YR 5/4), free, thick, unequal, slightly umbo. Stipe 79 mm × 8.5 mm, central, clavate, slightly wider at the base, fibrous, with longitudinal brownish (5.0YR 4/10) ciliate stripes, denser toward the base. Spore print unknown.

Basidiospores [60, 1, 1] (7.0)7.5–8.0(9.0) × 5.0–6.0 μm, avl = 8.0 μm, avw = 5.5 μm, Q = 1.16–1.50(1.60), Qm = 1.45 ± 0.10, broadly elliptical, oval to ovoid, slightly pinkish, slightly thick-walled, smooth, non-dextrinoid. Basidia 24–33 × 7–11(12) μm, clavate, 4-sterigmate, sterigmate short, less than 1 μm, thin-walled, and hyaline in KOH. Pleurocystidia 49–75(78) × (13)15–26 μm, mass, scattered, fusiform or narrowly fusiform to narrowly utriform, with 2–4 apical hooks (commonly entire), without small lateral hooks, hyaline, smooth, neck thickness up to 2–3 μm. Intermediate cystidia fusiform, poke-shaped, with pointed or flatter apices, without apical hooks, smaller than the pleurocystidia, thick-walled, hyaline. Cheilocystidia 38–60 × (11)12–15 μm, crowded, grouped to clustered, clavate or narrowly clavate to capitate, with obtuse at apices, thick-wall, and hyaline in KOH. Lamellar edge sterile. Pileipellis a cutis, with terminal elements 80–146 × 6–18 μm, individual elements cylindrical, some strongly tapering towards apex, mostly filled with brown intracellular pigment, thin-walled, smooth. Stipitipellis a cutis, hyphae 5–25 μm wide, cylindrical, hyaline or brown intracellular pigment, smooth, and thin-walled. Clamp connections absent in all tissues.

**Ecology.** Solitary or gregarious, growing on decayed wood (*Betula, Umbellularia, Populus*) or the humus layer under hardwoods or conifers in summer.

**Distribution.** Canada, Russia, USA, Turkey (Justo et al., 2014; Kaygusuz et al., 2021), and China (Xinjiang Uygur Autonomous Region)

**Specimens examined.** CHINA. Xinjiang Uygur Autonomous Region, Ili Kazakh Autonomous Prefecture, Zhaosu County, Shuimogou in Regiment 74, 43°12′50.69″N, 80°13′18.44″E, ASL 1764 m, 1 August 2021, Z.X. Qi, D.M. Wu, N. Gao & Y. Wang, HMJAU 60206 (OM991893, OM943513).

Note. *Pluteus brunneidiscus* and *P. washingtonensis* Murrill were first reported from USA (Murrill, 1917). However, Singer (1956), and Banerjee & Sundberg (1995) suggested that these two species may be the same. Singer (1986) cited *P. washingtonensis* as “probably conspecific with *P. brunneidiscus*” and with only one difference in the size of spores; besides, the spores of *P. washingtonensis* (about 6.5–9.6 × 5.3–7.1 µm) are slightly larger. Banerjee & Sundberg (1995) described the terminal elements on the pileipellis of *P. brunneidiscus* and *P. washingtonensis* as “versiform.” However, Justo & Castro (2007a) believe that the terminal elements were difficult to observe in the type collections. In modern collections, the shape of these elements was variable within the same basidiocarp and with the same range of variation observed. Therefore, they treated *P. washingtonensis* as synonymous with *P. brunneidiscus*. *P. brunneidiscus* in Europe was firstly described by Justo & Castro (2007a). In our study, *P. brunneidiscus* is reported as a new record in China.

In our phylogenetic analysis, *P. brunneidiscus* gathered into sect. *Pluteus*, with two other species—*P. shikae* Justo and E.F., *P. kovalenkoi* E.F.—in clade brunneidiscus. These three species can be distinguished from molecular data. Morphologically, many of their characters are rather variable, such as basidiospores and pleurocystidia with developed apical hooks. However, *P. kovalenkoi* can also be distinguished from *P. brunneidiscus* by the shape of pleurocystidia (Justo et al., 2014). We also compared other characters, as detailed in Table 2 (Justo et al., 2014; Kaygusuz et al., 2021).

**Table 2:** Morphological comparisons of brunneidiscus clade of *Pluteus brunneidiscus, P. shikae*, and *P. kovalenkoi*.

	*Pluteus brunneidiscus*	*Pluteus brunneidiscus* in China	*Pluteus shikae*	*Pluteus kovalenkoi*
Pileus	30–70 mm, hemispherical or campanulate to convex or plano-convex, smooth or fibrillose, brown to pure white, margin striate	68 mm, spreading to partially collapsed, shiny and smooth, grayish brown to brownish brown	20–50 mm, hemispherical or campanulate to convex or plano-convex, smooth, brown, margin striate	37–49 mm, obtusely campanulate to convex or plano-convex, smooth, fibrillose, brown or gray-brown, margin striate
Lamellae	crowded, free, ventricose, 7 mm broad, white to pink, flocculose edges	close, free, dirty white to dark brown, thick, unequal, ventricose	crowded, free, ventricose, 6 mm broad, white to pink, flocculose edges	crowded, free, slightly ventricose, 5 mm broad, white-cream to pink, concolorous edges
Stipe	30–80 × 3–7 mm, cylindrical, white, smooth or with longitudinal brown or gray-brown fibrils	79 × 8.5 mm, central, clavate, wider at the base, fibrous, with longitudinal brownish ciliate stripes	30–65(–70) × 3–6 mm, cylindrical, white, smooth or with longitudinal brown or gray-brown fibrils	70–80 × 5–6 mm, cylindrical, white or white-cream, glabrous or slightly pruinose
Context	white	not recorded	white	white
Spore print, Taste, Smell	Taste similar to smell or indistinct, Spore print pink to pinkish brown.	not recorded	not recorded	not recorded
Basidiospores	6.5–9.6(–10.5) × (4.5–)5.0–7.1 μm, slightly constricted in the middle	(7.0)7.5–8.0(9.0) × 5.0–6.0 μm, broadly elliptical, oval to ovoid,pinkish, smooth, non-dextrinoid	5.5–8.0 × (3.5–)4.0–5.5(–6.0) μm, slightly constricted in the middle	(7.3–)7.6–9.0(–9.5) × 4.6–5.8 μm, constricted in the middle
Basidia	15–28 × 6–12 μm, tetrasterigmate, clavate, some with median constriction	24–33 × 7–11(12) μm, tetrasterigmate, clavate	18–27 × 6–10 μm, tetrasterigmate, clavate, some with median constriction	18–30 × 5.5–8 μm, tetrasterigmate, constricted in the middle
Pleurocystidia	50–100 × 12–24(–30) μm, fusiform to narrowly utriform, 2–4 apical hooks, 3 μm thick-walled	49–75(78) × (13)15–26 μm, fusiform or narrowly fusiform to narrowly utriform, with 2–4 apical hooks (commonly entire), 2–3 μm thick-wall	60–97 × 12–22(–29) μm, fusiform to utriform, 2–4 apical hooks, sometimes lateral hooks, 3 μm thick-walled	70–90 × 10–25 μm, fusiform or utriform, 2–3 apical hooks, 2.7 μm thick-walled
Cheilocystidia	30–68 × 12–22 μm, clavate to spheropedunculate, hyaline, thin-walled	38–60 × (11)12–15 μm, crowded, grouped to clustered, clavate or narrowly clavate to capitate, with obtuse at apices	(27–)34–55 (65) × 12–20(–29) μm, clavate, hyaline, thin-walled	30–55 × 13–23 μm, clavate to utriform, hyaline, thin-walled
Intermediate cystidia	irregularly shaped, thin-walled	fusiform, poke-shaped, with pointed or flatter apices, smaller than the pleurocystidia, thick-walled	irregularly shaped, thin-walled	inflated fusiform, thin-walled
Lamellar edge	Lamellar edge sterile	Lamellar edge sterile	Lamellar edge sterile	Lamellar edges sterile
Pileipellis a cutis	80–146 × 8–15 μm, cylindrical, strongly tapering towards apex, brown intracellular pigment, thin-smooth walled	80–146 × 6–18 μm, individual elements cylindrical, tapering towards apex, filled with brown intracellular pigment, thin-smooth walled	90–150 × 7–17 μm, cylindrical, strongly tapering towards apex, brown intracellular pigment, thin-smooth walled	70–110 × 12–20 μm, cylindrical or slightly inflated, tapering towards apex, hyaline or pale yellow-brown intracellular pigment, thin-walled
Stipitipellis a cutis	5–25 μm wide, cylindrical, hyaline or brown pigment, thin-smooth walled	6–23 μm wide, cylindrical, hyaline or brown intracellular pigment, thin-smooth walled	5–20 μm wide, cylindrical, hyaline or brown pigment, thin-smooth walled	7–20 μm wide, cylindrical, hyaline or with yellow-brown intracellular pigment, thin-smooth walled
Clamp-connections	common and readily seen on pileipellis hyphae	absent in all tissues	common and readily seen on pileipellis hyphae	present on pileipellis hyphae
Ecolog	Solitary or gregarious	Solitary	Solitary or subgregarious	Subgregarious
Habit	growing on decayed wood of hardwoods or on the humus layer under hardwoods or conifers	growing on decayed wood of conifers in summer	growing on well-decayed wood of hardwoods, in hardwood-dominated or mixed forests	growing on well-decayed wood of conifers
Distribution	Russian, America, Turkey	China	Japan, Russian	Russian

The type specimen of *P. brunneidiscus* (Murrill, 1917) collected in Connecticut has a high similarity growth environment to our specimens, both in a temperate climate and autumn. We provide a detailed description of the morphological characteristics of *P. brunneidiscus*, which enriches Murrill’s (Murrill, 1917) report, such as the color variation of the pileus and the size range of the spores. At the same time, there are some differences. For example, the pileus of margin of our specimens has slightly sticky umbilical protrusions. They sometimes have translucent stripes at the pileus margin, while clamp connections are absent.

*Pluteus hongoi* Singer, Fieldiana, Bot. 21: 95 (1989).

Syn.: *P. major* Singer, Fieldiana, Bot. 21: 96 (1989);

Syn.: *P. albineus* Bonnard, Mycol. helv. 11(2): 131 (2001);

Syn.: *P. nothopellitus* Justo & M.L. Castro, Mycotaxon 102: 222 (2007).

(Figs. 4H–4I and 7)

**Figure 7:**
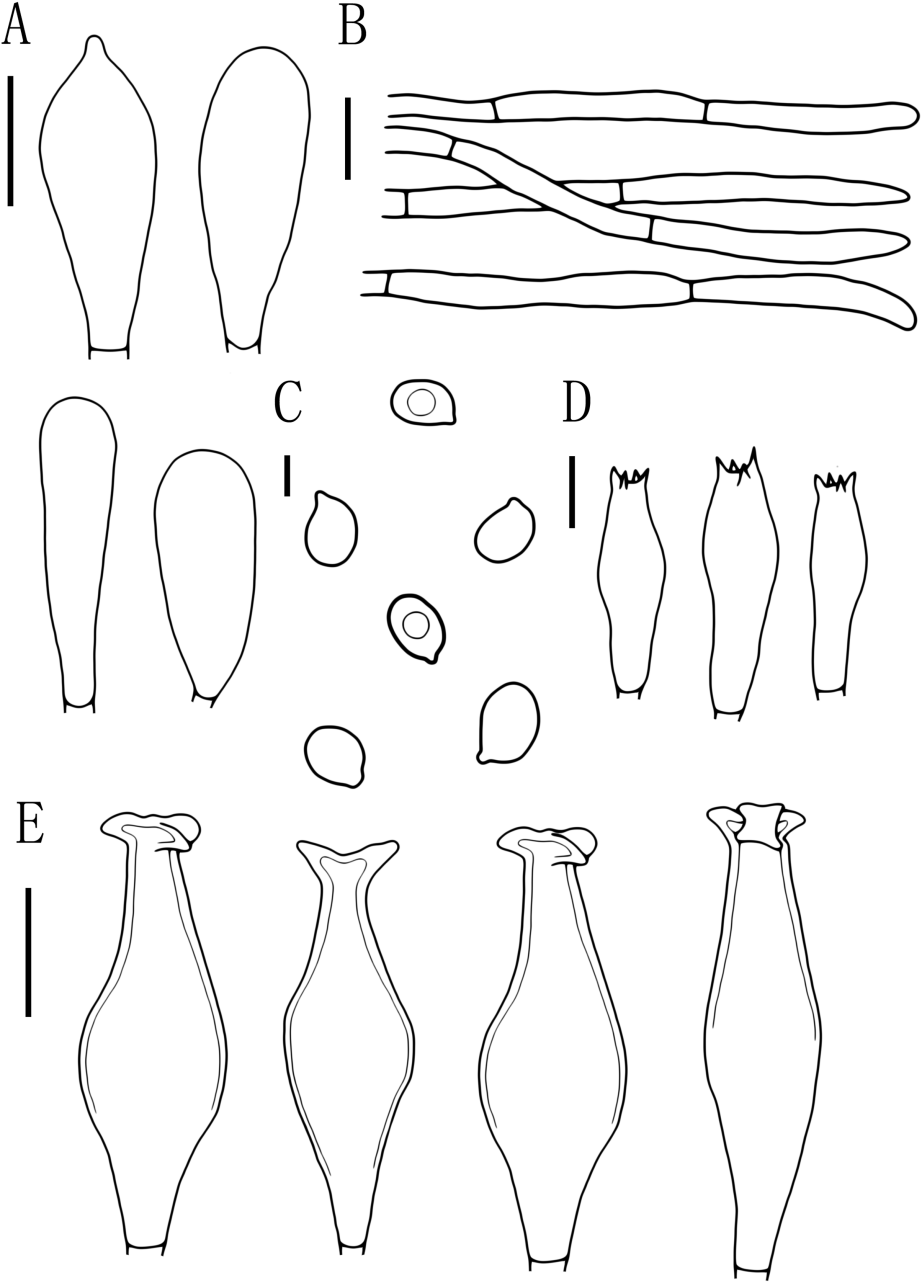
Microscopic features of *Pluteus hongoi*. (A) Cheilocystidia. (B) Pileipellis. (C) Basidiospores. (D) Basidia. (E) Pleurocystidia. Scalebars: (C) 5 μm; (D) 10 μm; (A, B, E) 20 μm.

**Description**

Basidiomata medium-sized. Pileus about 36.6 mm broad, flattened hemispherical, surface smooth and shiny, dark brown at the disc (7.5YR 4/2-7.5YR 6/2), becoming lighter toward the margin, margin transversely folded. Lamellae close, free, dense, folded, gray-white to earthy yellow (7.5YR 9/6-7.5YR 9/4), and slightly ventricose. Stipe 40 mm × 5.2 mm, central, subterete, fibrous, slightly expanded downward, white (7.5YR 9/2), smooth, with white longitudinally ciliate at the base. Spore print unknown.

Basidiospores [60, 1, 1] (7.0)8.0–9.5(10.0) × 5.5–6.5 μm, avl = 9.0 μm, avw = 6.0 μm, Q = 1.33–1.60(1.66), Qm = 1.50 ± 0.10, oval, pink, thin-walled, smooth, non-dextrinoid. Basidia 26–38 × 8–11 μm, clavate, usually 4-sterigmate, thin-walled, and hyaline in KOH. Pleurocystidia 55–83 × 14–23 μm, crowded, covering the whole side of the lamellae, flask-shaped to fusiform with 2–5 apical hooks (usually bifid), thick-walled, neck thickness up to 2–3 μm, hyaline in KOH. Cheilocystidia 29–57 × 12–20 μm, abundant, clavate or broad clavate to capitate, obtuse apices, hyaline, thick-walled. Pileipellis a cutis, with terminal elements 69–153 × 5–17 μm; individual elements cylindrical, some strongly tapering towards apex, mostly filled with brown intracellular pigment, smooth, and thin-walled. Stipitipellis a cutis, hyphae 5–19 μm wide, cylindrical, hyaline or brown intracellular pigment, smooth, and thin-walled. Clamp connections absent in all tissues.

**Ecology.** Solitary or scattered on rotten wood in mixed forests in summer and autumn.

**Distribution.** Japan, Spain, Russia, USA, Germany, Czech Republic, Slovakia, Republic of Korea, Turkey (Justo et al., 2014; Ševčíková, 2016; Kaygusuz et al., 2021), and China (Heilongjiang, Sichuan, Xinjiang Uygur Autonomous Region) (Xu, 2016).

**Specimen examined.** CHINA. Xinjiang Uygur Autonomous Region, Aletai Region, Aletai City, Wolong Bay in Kanas, 48°65′77.01″N, 87°03′82.11″E, ASL1333 m, 16 September 2021, Z.X. Qi, D.M. Wu, N. Gao & B.K. Cui, HMJAU60205 (OM302007).

Note. Singer (1989) first described *P. hongoi* and *P. major* Singer. Later, *P. hongoi* is recognized by Justo et al. (2014), because the original description of this taxon is more complete than *P. major*. Compared with our specimen, the type specimens (Singer, 1989) have smaller basidiospores, and the apical hook’s structure was often dehiscent.

Justo et al. (2014) studied the species belong to section *Pluteus* in the Holarctic region. They combined the molecular data based on type specimens of *P. major*, *P. albineus* Bonnard (Bonnard, 2001), and *P. nothopellitus* Justo & Castro (2007b) indicate that all these species represent different morphological variants of *P. hongoi*.

*Pluteus hongoi* was easily confused with *P. cervinus*. The pileus of *P. cervinus* (Murrill, 1917; Justo et al., 2014) was very variable in colors (brown, gray-brown, orange-brown, white), aspect of the pileus (with or without conspicuous squamules and radial fibrils) the stipe often had longitudinal and brown, or gray-brown fibers or scales, and the apical hooks structure (commonly entire) of the pleurocystidia. However, these features are variable and cannot be easily expressed on a basidiocarp (Justo et al., 2014). But they can be distinguished by sequences analyses.

**Key to the reported species of *Pluteus* in Xinjiang Uyghur Autonomous Region**

1. Metuloid cystidia...............................................................................................................................................2
- Non-metuloid cystidia.........................................................................................................................................5
2. Pleurocystidia apical hooks commonly entire...................................................................................................3
- Pleurocystidia apical hooks usually bifid..............................................................................................***P. hongoi***
3. With intermediate cystidia.................................................................................................................................4
- Without intermediate cystidia...............................................................................................................***P. pellitus***
4. Stipe has longitudinal brown stripes, basidiospores (7.0)7.5–8.0(9.0) × 5.0–6.0, pleurocystidia 2–4 apical hooks..........................***P. brunneidiscus***
- Stipe has continuous longitudinal brown fibrillose, basidiospores 5.5-8(-9) × 4.5-7, pleurocystidia 3–5 apical hooks....................................***P. cervinus***
5. Pileipellis a trichoderm.....................................................................................................................................6
- Pileipellis a non-trichoderm................................................................................................................................7
6. Pileus black-brown.........................................................................................................................***P. umbrosus***
- Pileus golden to dull or brownish yellow............................................................................................***P. leoninus***
7. Pleurocystidia and cheilocystidia with tapered apices....................................................................***P. thomsonii***
- Pleurocystidia and cheilocystidia without tapered apices................................................................***P. aletaiensis***

## Discussion

In this study, one new species, *Pluteus aletaiensis*, a new record species from China, *P. brunneidiscus*, and a new record species from Xinjiang Uyghur Autonomous Region, China, *P. hongoi*, were discovered in Xinjiang Uyghur Autonomous Region based on morphological studies and phylogenetic analyses.

*P. brunneidiscus* without clamp connections is very interesting. Clamp connections are an essential discriminating character in the brunneidiscus clade, and in the articles of Justo & Castro (2007a), Justo et al. (2014), and Kaygusuz et al. (2021), *P. brunneidiscus* are present clamp connections; however, our specimen does not have this feature in all tissues, which is unusual and could be caused by the environmental specificity here, and it was growing on a mainly decayed *Picea schrenkiana* Fischet Mey. stump.

Most species of the genus *Pluteus* are saprophytic on trees (conifers or angiosperms). Some of them are also indirectly associated with trees, *e.g., P. cervinus*, *P. hongoi*, *P. elaphinus* Justo, *P. petasatus* (Fr.) Gillet, *P. pellitus* (Pers.) P. Kumm., *P. salicinus* (Pers.) P. Kumm., *P. fulvibadius*, *P. amphicystis* Singer, and *P. americanus* (P. Banerjee & Sundb.) Justo, E.F. Malysheva & Minnis, which are always found to grow on angiosperm wood or in the humus layer without apparent connection to wood (mostly *P. hongoi* and *P. petasatus*) or piles of woodchips (especially *P. petasatus* and *P. pellitus*) (Justo et al., 2014). However, there is also a small percentage of species that are only found on the ground, without association with trees. *e.g., P. ephebeus* (Fr.) Gillet, *P. fenghuangensis* Z.S. Bi, *P. nankungensis* Z.S. Bi & T.H. Li, and *P. aletaiensis*. In summary, there are some adaptation mechanisms between these species and trees or ecology, *e.g.*, growing on decaying material of specific tree species or decaying material in all tree species, growing on decaying material of live trees/fallen wood/dead trees/stumps, etc., indirectly having some association with trees, growing on the ground/humus, not associated with trees. In the future, subsequent studies of *Pluteus* will require additional data to further support it.

## Additional Information and Declarations

### DNA Deposition

The following information was supplied regarding the deposition of DNA sequences: The ITS sequences are available at NCBI: OM991943, OM992247, OM992249, OM991893, OM943513, OM992252, OM302007. Sequences are provided for review at https://doi.org/10.6084/m9.figshare.19387640.

### Data Availability

The following information was supplied regarding data availability: Pluteus aletaiensis Z. X. QI, B. Zhang & Y. Li, sp. nov. MycoBank No: 840297 The specimen was deposited in the Herbarium of Mycology of Jilin Agricultural University (HMJAU) that is one of the four major public mycological herbaria in China. The specimen numbers are: HMJAU60205, HMJAU60206, HMJAU60207, HMJAU60208, HMJAU60209. The TEF1α GenBank accession numbers for HMJAU 60207, HMJAU 60208, and HMJAU 60209 are available at NCBI: OP573273, OP573274, and OP573275.

### New Species Registration

The following information was supplied regarding the registration of a newly described species: Pluteus aletaiensis Z. X. QI, B. Zhang & Y. Li, sp. nov. MycoBank No: 840297.
